# Bioactive Compounds and Related Food-Medicine Homology Potential of *Prinsepia utilis* Seed Oil

**DOI:** 10.3390/molecules31101700

**Published:** 2026-05-17

**Authors:** Changran Li, Ruyi He, Xiaoya Yin, Angkhana Inta, Maroof Ali, Lu Gao, Ruyu Yao, Lixin Yang

**Affiliations:** 1 School of Ethnic Medicine, Yunnan Minzu University, Kunming 650201, China; 2 Key Laboratory of Economic Plants and Biotechnology, Kunming Institute of Botany, Chinese Academy of Sciences, Kunming 650201, China; 3 Department of Biology, Faculty of Science, Chiang Mai University, Chiang Mai 50200, Thailand; 4 Center for Integrative Conservation, Xishuangbanna Tropical Botanical Garden, Chinese Academy of Sciences, Menglun, Mengla 666303, China; 5 Yunnan International Joint Laboratory of Health Plant Resources Development & Yunnan Key Laboratory for Wild Plant Resources, Kunming Institute of Botany, Chinese Academy of Sciences, Kunming 650201, China; 6 Yunnan International Joint Laboratory of Southeast Asia Biodiversity Conservation, Menglun 666303, China; 7 Bio-Innovation Center of DR PLANT, Kunming Institute of Botany, Chinese Academy of Sciences, Kunming 650201, China

**Keywords:** seed oil, *Prinsepia utilis*, core compounds, antioxidant, anti-aging activities, skin repair activity

## Abstract

The seed oil of *Prinsepia utilis* is a traditional food-medicine homology used by the Naxi people in Yunnan Province of the Himalayan region. This study explored the potential applications of food-medicine homology in health and wellness. Using untargeted metabolomics, we compared the metabolite profiles of the subcritical extraction method (edible oil as crude oil) (CO) and its improved processing method (medicinal oil as refined oil) (RO) extracted from *P. utilis* seeds by UPLC–QTOF MS/MS and evaluated their in vivo and in vitro activities. We screened 14 discriminatory metabolites and performed their tentative annotation, including fatty acids, terpenoids, steroids, and quinones. Furthermore, we quantified 12 bioactive compounds in CO samples via targeted chromatographic analysis, which support the promising food-medicine homology applications of the oil. RO exhibited potent antioxidant activity (DPPH radical scavenging rate of 79.7%, representing 65.7% increase over CO), with an ABTS^+^ radical scavenging rate of 95.8% (77.1% improvement over CO). The hyaluronidase inhibition rate for RO was 43.4%, whilst that for CO was 30.8%; the elastase inhibition rate for RO was 69.8%, whilst that for CO was 59.8%, and promoted zebrafish fin regeneration by 15% at 3% concentration. Our results validated the seed oil of *P. utilis* as a traditional food and its antioxidants, anti-aging, demonstrating that the CO processing method is able to meet with medicine and food homology in health, and the RO processing method may satisfy skin care function. These findings highlight the potential applications of *P. utilis* seed oil for food-medicine homology in health and wellness properties.

## 1. Introduction

Traditional medicinal and edible cultures of ethnic groups in the world not only provide significant information for the sustainable utilization and protection of medicinal and edible plant resources. At the same time, it also provides an important knowledge prototype for the research and exploitation of edible and medicinal food resources. The plants with traditional classical usage are being primarily studied [1]. However, exploring the properties of plant food that contribute to healthcare is still needed to further develop [2]. High-altitude environments are typified by hypoxia, intense UV radiation, and substantial diurnal temperature variations. Through long-term adaptive evolution, medicinal and edible plants native to these regions display elevated biosynthesis of secondary metabolites [3]. For example, in response to high-altitude UV radiation, key enzymes in the phenylpropanoid pathway accumulate in such plants, increasing the accumulation of polyphenolic compounds [4]. These phenolic metabolites have notable antioxidant and anti-inflammatory properties [5].

The seed oil of plants is rich in primary and secondary metabolites, attracting interest for use in traditional foods, pharmaceuticals, and cosmetic products [6]. *Prinsepia utilis* Royle, cultivated in Naxi communities of the eastern Himalayas, Yunnan,China, a medicinal and economically important highland woody oilseed plant, is a deciduous thorny shrub belonging to the Rosaceae family. Distributed in areas of southwestern China, particularly in northwest Yunnan in the eastern Himalayan region, *P. utilis* grows at altitudes from 1600 m to 3000 m [7,8]. Its traditional uses in food and medicine were first documented in the classical medical manuscripts Dian Nan Herb and Yu Long Herb over 500 years ago. These classic manuscripts indicate that tender leaves of *P. utilis* were chewed thoroughly and applied topically as a poultice. In traditional medicine, extracts from its tender stems, leaves, and fruits are recognized for their anti-aging effects and are widely used to treat various skin disorders. The tender leaves of *P. utilis* have a bitter taste and are used to treat wounds on the skin and reduce swelling [9,10]. Traditionally, ethnic groups in the high-altitude regions of northwestern Yunnan extract oil from the seeds, tender leaves, roots, stems, flowers, and fruits for food and medicinal use [11]. The oil is applied topically to treat skin conditions such as chapping, sunburn, frostbite, dryness, itching, and infantile eczema, and is also used for culinary purposes.

Previous studies have reported multiple beneficial properties associated with *P. utilis* seed oil, including antioxidant [12], antibacterial [13,14], and anti-inflammatory activities [13]. *P. utilis* seed oil is rich in primary metabolites, including oleic acid and linoleic acid, which possess notable anti-inflammatory activity, highlighting its promising health care benefits. In addition, *P. utilis* seed oil is rich in secondary metabolites, including sterols (β-sitosterol, cholesterol, campesterol, and stigmasterol), which confer health benefits, contribute to cosmetic activity, and enhance functional foods, providing protection against UV light and possessing anti-aging and wound healing properties [14].

Several bioactive compounds in *P. utilis* seed oil have been identified as suitable for developing health products [15]. A clinical study of a skin cream containing *P. utilis* seed oil demonstrated its safety and efficacy in treating skin disorders [16]. Furthermore, this oil is rich in tocopherols, especially γ-tocopherol, which act as antioxidants and protect oils from degradation [17], as well as phenolic compounds, particularly gallic acid [18]. The antioxidant activity of *P. utilis* oil depends on its source and production method [19]. Notably, conventional industrial refining of vegetable oils typically leads to reduced antioxidant capacity by removing phenols, tocopherols, and other bioactive minor components. Our hypothesis is that subcritical extraction and targeted refining processes can selectively enrich specific functional components while removing non-lipid impurities, thereby potentially enhancing the overall biological activity of the oil. However, the metabolites underlying the beneficial properties of *P. utilis* seed oil remain unclear based on subcritical extraction (edible oil as crude oil) (CO) and its improved processing method (medicine oil as refined oil) (RO). Here, we aimed to (1) analyze the compounds of *P. utilis* seed oil using UPLC–QTOF MS/MS, (2) investigate the in vitro and in vivo biological activities of the oil using antioxidant assays, hyaluronidase (HAase) and elastase-inhibitory assays, and a zebrafish restoration model; and (3) evaluate the effects of two oil processing methods on the biological activity and metabolite profiles of seed oil from this traditional food and medicinal plant.

## 2. Results and Discussion

### 2.1. Characterization of Differential Metabolites in P. utilis Seed Oil

**Figure 1:**
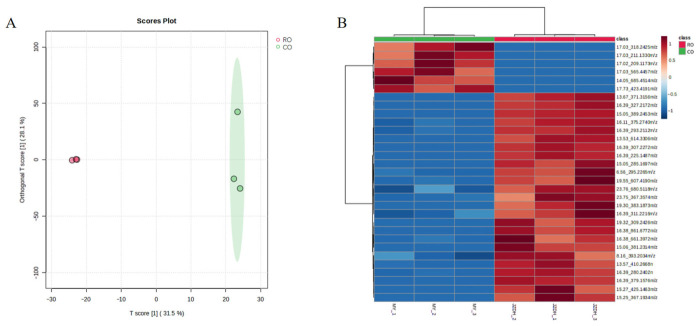
Metabolomic profiling of CO and RO from *P. utilis* seeds. (**A**) Orthogonal partial least squares-discriminant analysis (OPLS-DA) score plot showing clear separation between the two groups. (**B**) Heatmap of differential metabolite features (VIP > 1, *p* < 0.05) between CO and RO, displaying relative abundance patterns across samples.

Non-targeted UPLC-QTOF MS/MS metabolomics was employed to characterize the metabolic differences between CO and RO from *Prinsepia utilis* seeds. Orthogonal partial least squares-discriminant analysis (OPLS-DA) yielded a clear separation between CO and RO (Figure 1A), with model parameters of *R*^2^*X* = 0.315, *R*^2^*Y* = 0.964, and *Q*^2^(cum) = 0.669, indicating distinct metabolic profiles shaped by the refining process. A total of differential metabolite features with variable importance in projection (VIP) > 1 and *p* < 0.05 were screened, and their abundance patterns are visualized in the heatmap (Figure 1B).

These results indicate that different processing methods have a significant impact on the enrichment of various components in *P. utilis* seed oil, as well as on the stability of certain functional components. For annotation accuracy, only metabolites with confirmed plant-derived origins were tentatively annotated (Supplementary Table S1). These metabolites spanned multiple chemical classes, including long-chain fatty acids and their oxidized derivatives (e.g., 17-octadecynoic acid, hydroxyoctadecadienoic acid, and octacosanoic acid), as well as a diverse array of terpenoids and phenolic-related constituents (e.g., sterebin D, petasin, vulgarole, and 4,5-dihydrovomifoliol). The presence of both lipid-derived compounds and specialized secondary metabolites among the significantly altered features underscores the multifaceted impact of the refining procedure on the chemical composition of *P. utilis* oil.

**Figure 2:**
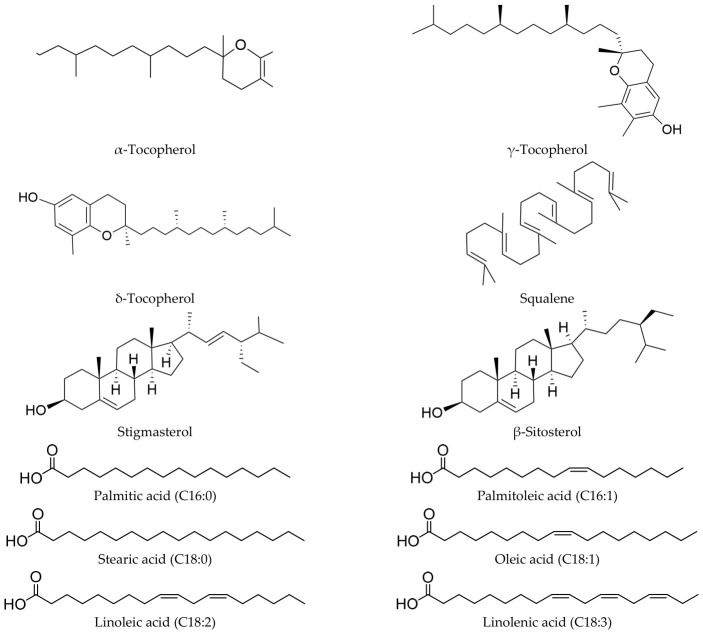
Structural formulae of the twelve compounds in the CO sample.

Notably, we identified 12 bioactive compounds in the CO sample and none in the RO sample, including tocopherols, squalene, sterols, and fatty acids. Specifically, we detected three tocopherol homologs (α-, γ-, and δ-tocopherol) (Supplementary Figure S1), one squalene (Supplementary Figure S2), two sterol homologs (stigmasterol and β-sitosterol) (Supplementary Figure S3), and six fatty acids (palmitic acid (C16:0), palmitoleic acid (C16:1), stearic acid (C18:0), oleic acid (C18:1), linoleic acid (C18:2), and linolenic acid (C18:3)) (Supplementary Figure S4). Their structures are shown in Figure 2. and their biological activities are as follows: the three tocopherols have antioxidant [20], antibacterial, and anti-inflammatory properties [21,22]; squalene shows immunomodulatory, hypolipidemic, and antitumor effects [23]; the two sterols exhibit anti-inflammatory [21] and cholesterol-lowering activities [24]; the six fatty acids provide energy and display anti-inflammatory effects (C16:0, C18:0) and unsaturated acids (C16:1, C18:1, C18:3) or contribute antioxidant [25] and anti-inflammatory effects (C16:1, C18:1, C18:3) [26]. These findings highlight the great potential of seed oil from *P. utilis* as a CO made for medicine and food of homological applications.

### 2.2. Antioxidant Activities

**Figure 3:**
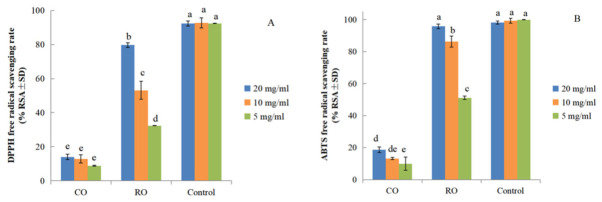
Antioxidant capacity of CO and RO prepared from *P. utilis* seeds (**A**) DPPH radical scavenging activity and (**B**) ABTS^+^ radical scavenging activity. Values are presented as means ± standard deviation (SD; *n* = 3). Statistical significance was determined using a two-way ANOVA followed by a post hoc Duncan test. Different lowercase letters indicate significant differences (*p* < 0.05).

A chemical compound has antioxidant activity when it protects biological systems from damage caused by oxidative stress. Antioxidants are essential for preserving biological systems from harm induced by free radicals [27]. The stable radical DPPH is widely used to assess the primary antioxidant activity of pure antioxidants, plant extracts, and raw materials in cosmetics [28]. The assay is based on the reduction of DPPH radicals in absolute ethanol, which is indicated by a decrease in absorbance at 517 nm. In this study, we evaluated the antioxidant activity of CO and RO samples using the DPPH and ABTS^+^ free radical scavenging assays (Figure 3).

Both CO and RO samples exhibited dose-dependent DPPH free radical scavenging activity, with scavenging rates increasing significantly as concentrations rose from 5 mg/mL to 20 mg/mL (Figure 3A). Notably, RO samples consistently showed higher DPPH scavenging activity than CO samples across all tested concentrations, with the highest activity of 79.7 ± 2.3% observed in RO at 20 mg/mL. Two-way ANOVA (Table S2) showed significant main effects of sample type and concentration on DPPH radical scavenging rate (*p* < 0.001 for both), as well as a significant sample × concentration interaction (*F* = 96.315, *p* < 0.001, partial η^2^ = 0.955), indicating distinct concentration-dependent activity patterns between CO and RO. The main effects were extremely large within the fitted model (sample type: *F* = 2764.966, *p* < 0.001, partial η^2^ = 0.997; concentration: *F* = 130.437, *p* < 0.001, partial η^2^ = 0.935). Duncan’s post hoc test indicated that RO samples had significantly higher DPPH scavenging activity than CO samples at all tested concentrations (*p* < 0.05).

For ABTS^+^ radical scavenging activity (Figure 3B), RO samples also exhibited superior activity compared to CO samples at each concentration. At 20 mg/mL, RO samples achieved the highest ABTS^+^ scavenging rate of 95.8 ± 0.6%, which was comparable to the positive control (98.2 ± 4.0%). Two-way ANOVA (Table S3) confirmed significant main effects of sample type and concentration on ABTS^+^ scavenging rate (*p* < 0.001 for both), alongside a significant sample × concentration interaction (*F* = 148.025, *p* < 0.001, partial η^2^ = 0.970), consistent with the DPPH results. The main effects were also extremely large in this model (sample type: *F* = 5202.517, *p* < 0.001, partial η^2^ = 0.998, concentration: *F* = 209.371, *p* < 0.001, partial η^2^ = 0.959). Duncan’s post hoc test further verified that RO samples had significantly higher ABTS^+^ activity than CO samples at all concentrations (*p* < 0.05).

### 2.3. Activity of Anti-Aging Enzymes

**Figure 4:**
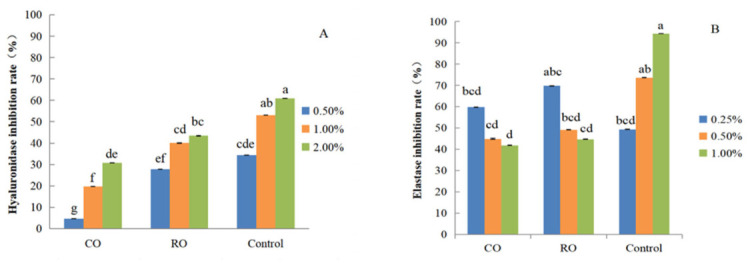
Hyaluronidase and elastase inhibition assays using CO and RO samples from *P. utilis* seeds. (**A**) Hyaluronidase inhibition assay. Dipotassium glycyrrhizinate was used as a positive control. (**B**) Elastase inhibition assay. EGCG was used as a positive control. Values are presented as means ± SD (*n* = 3). Statistical significance was evaluated using a two-way ANOVA followed by post hoc Duncan’s test. Different lowercase letters denote significant differences at *p* < 0.05.

Upon stimulation by allergens, hyaluronidase (HAase), an enzyme responsible for the depolymerization of hyaluronic acid (HA), is excessively activated, breaking down HA and resulting in the deregulation of skin homeostasis and the aggravation of inflammatory and allergic states [29]. Consequently, the skin loses elasticity and integrity, wrinkles appear, and the visible signs of aging accelerate [30]. Inhibiting human elastase is necessary to prevent its excessive activity from over-degrading elastin, which would otherwise lead to issues like skin sagging or reduced blood vessel elasticity [31]. We examined the potential anti-aging activity of *P. utilis* seed oil by measuring its ability to inhibit HAase and elastase activity. CO and RO samples exhibited strong HAase inhibition. At the same concentration, the inhibition rate of HAase activity by RO samples was significantly higher than that by CO samples (*p* < 0.05) (Figure 4).

When the concentration of CO and RO samples increased from 0.5% (*v*/*v*) to 2.0% (*v*/*v*), HAase inhibition correspondingly increased, with the RO samples reaching the maximum HAase inhibition (43.4 ± 2.5%) at the highest concentration tested (2.0%). Two-way ANOVA (Table S4) revealed significant main effects of both sample type and concentration on HAase inhibition rate (all *p* < 0.001). A strong sample × concentration interaction was also observed (*F* = 152.860, *p* < 0.001, partial η^2^ = 0.944), indicating that the magnitude of HAase inhibition by CO and RO varied with concentration. Duncan’s post hoc test confirmed that RO samples displayed significantly higher HAase inhibition activity than CO samples at each concentration level (*p* < 0.05).

We performed similar assays to quantify the extent of elastase inhibition imposed by CO and RO samples. At the lowest concentration tested (0.25% [*v*/*v*]), the elastase inhibition rate by RO samples was significantly higher than that obtained with CO samples (Figure 4). Increasing the amount of CO or RO samples in the reaction to 0.5% or 1% achieved a similarly high elastase inhibition rate (69.8 ± 2.5%), which was not significantly different from that obtained with 0.5% EGCG (positive control; 73.7 ± 5.6%). Two-way ANOVA (Table S5) demonstrated significant main effects of sample type and concentration on elastase inhibition rate (*p* < 0.001 for both). A significant sample × concentration interaction was detected (*F* = 3.053, *p* = 0.044, partial η^2^ = 0.404), demonstrating that the effect of sample type on elastase inhibition was concentration-dependent. In general, the enhanced inhibition of HAase and elastase activities by RO samples indicates that the properties of *P. utilis* seed oil are improved through the RO-made processing method.

These results demonstrate that the bioactive compounds in *P. utilis* seed oil could have applications in skincare. The skincare benefits of *P. utilis* seed oil, including its soothing and anti-inflammatory effects, have previously been mainly attributed to its sterols, based on empirical data and chemical composition [6]. Sterols may be promising contributors to the HAase and elastase-inhibitory effects observed in this study. Additionally, fatty acids, which are the main components of the oil, may also potential benifit to the elastase-inhibitory activity of *P. utilis* seed oil. Our phytochemical analysis tentatively annotated some relative compounds, such as campesterol glucoside, 17-octadecynoic acid, and achieved a synergistic effect as the principal discriminators between CO and RO samples (Supplementary Table S1). The higher relative abundance of these tentatively annotated compounds in RO samples is consistent with their enhanced anti-HAase and anti-elastase activities compared to CO samples.

### 2.4. In Vivo Repair Promotion Rates of Amputated Tail Fins in Zebrafish Embryos

**Figure 5:**
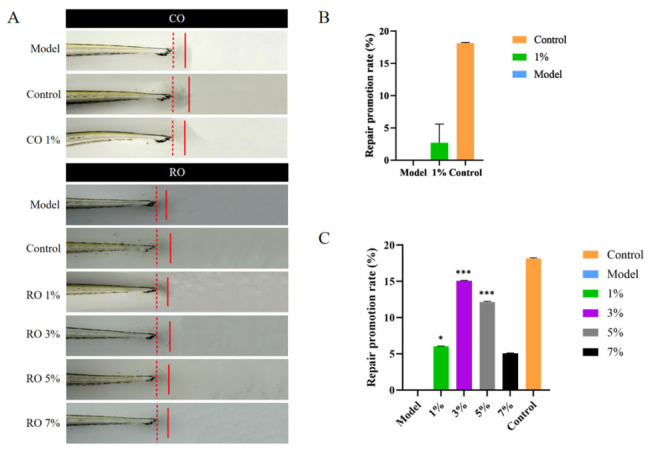
Effects of *P. utilis* seed oil extracts on caudal fin repair in zebrafish embryos (**A**) Representative photographs of caudal fin regeneration following treatment with CO and RO. The distance between the red dashed and solid lines indicates the repaired region of zebrafish embryonic caudal fins. (**B**) Repair promotion rate of zebrafish embryo caudal fins treated with 1% CO. (**C**) Repair promotion rate of zebrafish embryo caudal fins treated with 1%, 3%, 5%, and 7% RO. Statistical significance was evaluated using a one-way ANOVA. *, *p* < 0.05; ***, *p* < 0.001.

The main steps and principles of wound healing in zebrafish and humans are very similar, making zebrafish an effective model for detecting and screening human repair efficacy [32]. We inflicted full-thickness wounds to the caudal fins of zebrafish embryos to induce skin injury. We evaluated the ability of RO and CO samples to repair these amputated tail fins. When the RO sample concentrations were added to 1%, 3%, 5%, and 7%, the repair promotion rates of amputated tail fins were 6%, 15%, 12%, and 5%, respectively (Figure 5A (RO) and Figure 5C). These results indicate that three RO sample concentrations ranging from 1% to 5% significantly facilitated wound closure of the Zebrafish embryo. Adding 1% CO or 7% RO to Zebrafish embryos with amputated tail fins had a similarly low and significant effect on fin repair (Figure 5B,C). Additionally, we evaluated the toxicity of *P. utilis* seed oil on these zebrafish embryos. Even at the highest amount added of 7%, *P. utilis* seed oil did not adversely affect the embryonic development of zebrafish, suggesting that *P. utilis* seed oil has no apparent toxicity to zebrafish embryos. The result of this analysis indicates that RO has a promising application in cosmetics and pharmaceuticals.

## 3. Materials and Methods

### 3.1. Materials and Reagents

Seed pods of *P. utilis* plants were collected in October 2022 from Yongsheng County, Lijiang City, Yunnan, China. A voucher specimen (No. 1644011) was identified by Dr. Lixin Yang at the Herbarium in the Kunming Institute of Botany, Chinese Academy of Sciences. All samples were kept in the dark at room temperature (20–22 °C) until analysis.CBE-100L subcritical extraction equipment was purchased from Henan Subcritical Extraction Technology Co. (Anyang, China).

Methanol (HPLC grade), acetonitrile (HPLC grade), and dichloromethane (analytical grade) were purchased from Innochem Co., Ltd. (Beijing, China). α-Tocopherol (≥97%), γ-tocopherol (≥98%), squalene (≥98%), stigmasterol (≥95%), and β-sitosterol (≥98%) were purchased from Shanghai Yuanye Bio-Tech. Ltd. (Shanghai, China); δ-tocopherol (≥97%) was purchased from Shanghai Aladdin Biochemical Technology Co. (Shanghai, China). 1,1-Diphenyl-2-picrylhydrazyl (DPPH), 2,2′-azino-bis (3-ethylbenzothiazoline-6-sulfonic acid) (ABTS), hyaluronidase, and elastase were provided by Sigma-Aldrich (St. Louis, MO, USA). Epigallocatechin-3-gallate (EGCG), Tris, AAAPVN, acetylacetone, p-dimethylaminobenzaldehyde, anhydrous potassium acetate, ethanol (analytical grade), ascorbic acid, polysorbate 20, sodium hydroxide, potassium iodide, phenolphthalein, 2,6-di-tert-butyl-p-cresol (BHT), methyl tert-butyl ether, and dimethyl sulfoxide (DMSO, analytical grade) were obtained from Macklin Biochemical Technology Co., Ltd. (Shanghai, China). Before use, purified water was processed via a Milli-Q Integral 5 system (Millipore, Merck KGaA, Darmstadt, Germany). n-butane (BR): purchased from Shanghai McLean Biochemical Technology Co. (Shanghai, China). Working solutions were made each day. Unless otherwise specified, the reagents used in this study were analytically pure or chromatographically pure, and the water was grade 3 water as specified in GB/T 6682 [33].

### 3.2. Analysis of Compounds in P. utilis Seed Oil

#### 3.2.1. Sample Pretreatment

CO made by subcritical extraction: The seeds of *P. utilis* were softened at 40–60 °C for 40 min after removal of impurities and separation of shell and kernel, followed by flaking and drying at 45 °C to remove moisture. An appropriate quantity of the processed meal was accurately weighed, then loaded into a subcritical extraction tank. The feed inlet was subsequently sealed, and a vacuum pump was engaged to evacuate the tank until the internal pressure was reduced to below 0.1 MPa. Subsequently, n-butane in a subcritical state was introduced, with a liquid-to-solid ratio of 1:1 (mL:g). The extraction was carried out at 60 °C and 0.9 MPa for 30 min, and this process was repeated 4–5 times. Upon completion of extraction, the crude oil and n-butane mixture was transferred to a separation tank, where it was subjected to reduced pressure (to −0.07 MPa) at 45 °C for vaporization. The vaporized n-butane was compressed, liquefied, and recycled for repeated use. Following desolventization, the crude *P. utilis* seed oil was discharged from the system, with a final extraction yield of 18–20% (*w*/*w*, based on the dry weight of the processed meal).

RO made by optimal processing technique method: Crude oil (CO) was processed using patented technology (ZL 2024 2 0328799.6) combined with conventional production methods at a vegetable oil plant, undergoing five-stage refining to produce refined oil (RO). First, colloidal substances were removed through hydration degumming. Then, food-grade activated carbon was used for decolorization to eliminate pigments and odor precursors. Subsequently, acid removal occurred in a tandem alkali refining tank using a ratio of 60–70 kg of 12.5–13.5% sodium hydroxide solution per ton of oil. The acid-removed oil was filtered and temporarily stored. Finally, it underwent deodorization in a deodorization tank heated to 145–165 °C with dual heating elements, followed by cooling with chilled water to complete the refining process.

For each sample (CO and RO), 100 μL was mixed separately with 200 μL HPLC-grade methanol and 200 μL dichloromethane. After centrifugation, the supernatant was transferred to sample vials for testing. Three replicate samples were set up per group, totaling six samples to ensure testing accuracy. (Three replicate samples were set up per group, totaling six samples to ensure testing accuracy).

#### 3.2.2. UPLC–QTOF MS/MS Analysis

The CO and RO samples were sent to Beijing Yingnuo Kaisheng Technology Co. (Beijing, China)for UPLC–QTOF MS/MS analysis. The phytochemical profiles of the samples were determined using a Waters ACQUITY UPLC I-Class system (Milford, MA, USA). A 1.7 μm Waters ACQUITY UPLCr BEH C18 column was used for separation, with a binary gradient elution system made up of 0.1% (*v*/*v*) aqueous formic acid (A) and acetonitrile (B). The elution program was configured as follows: 0 min, 5% (*v*/*v*) B; 4 min, 10% (*v*/*v*) B; 21.5 min, 100% B; 23 min, 100% B; 25 min, 5% (*v*/*v*) B. Flow rate was kept at 0.4 mL/min, with the injection volume set to 2 μL. Temperatures for the column and sample tray were kept at 30 °C and 15 °C, respectively [34]. Settings for the mass spectrometer comprised a capillary voltage of 2.0 kV, a desolvation temperature of 450 °C, and a desolvation gas (N_2_) flow rate of 900 L/h. The mass scan range was set to 100–1200 *m*/*z*, utilizing the MSE collection mode and sensitivity mode. Data analysis was conducted using MassLynx 4.2 software for raw data collection and file formatting. Progenesis QI software v3.1 was used for peak alignment, identification, normalization, and deconvolution analysis of the original data. Metabolite tentative annotation was performed by matching accurate mass, molecular formula and MS/MS fragment ions against the Human Metabolome Database. Public spectral databases were used solely for tentative feature annotation of metabolites, and not to assert organism-specific or biosynthesis-specific origin of the annotated features. Metabolomic data were analyzed using the MetaboAnalyst R tools 4.3 to perform principal component analysis (PCA) and orthogonal partial least squares discriminant analysis (OPLS-DA), among other methods, to detect differences between experimental groups and assess intra-group reproducibility. Differential metabolite features were screened based on the criteria of variable importance in projection (VIP) ≥ 1 and *p* < 0.05.

#### 3.2.3. UPLC Analysis

Tocopherol was quantified in *P. utilis* seed oil according to GB 5009.82-2016 [35] “National Food Safety Standard for the Determination of Vitamins A, D, and E in Foods”. High-performance liquid chromatography (HPLC) conditions were as follows: a ZORBAX SB-C18 reversed-phase column (4.6 mm × 250 mm, 5 μm) was used, the column temperature was maintained at 30 °C, and the mobile phase was a methanol–water solution (98:2, *v*/*v*). Isocratic elution was applied, with a flow rate of 1.0 mL/min, UV detection at a wavelength of 294 nm, and an injection volume of 20 μL. The determination of squalene in *P. utilis* seed oil followed the protocol SN/T 4785-2017 [36] “Determination of squalene in exported vegetable oils”.

Quantitative analysis of sterols in *P. utilis* seed oil was performed according to the method of Yin et al. [37]. For sample treatment, 200 mg (accurate to 0.1 mg) of *P. utilis* seed oil was weighed in a 10 mL Polyvinyl Chloride Tube, and 2 mL of 2 mol/L potassium hydroxide-ethanol mixture was added. The mixture was vortexed for 2 min to ensure thorough mixing, followed by incubation in a 60 °C water bath for 1 h. At the end of the reaction, 2 mL of distilled water and 2 mL of n-hexane were added. After centrifugation (4000 rpm, 6 min, room temperature) and stratification, the supernatant was collected. This procedure was repeated three times. All supernatants were combined and rinsed with distilled water until neutral. The hexane layer was separated, and the organic phase was dried over anhydrous sodium sulfate before being adjusted to a final volume of 6.0 mL. A 3.0 mL aliquot of this extract was dried under a gentle nitrogen stream, reconstituted in 1.0 mL of acetonitrile, and filtered through a 0.22 μm membrane prior to liquid chromatography analysis. Conditions for HPLC were as follows: a ZORBAX SB-C18, reversed-phase column (4.6 mm × 250 mm, 5 μm) was used; the column temperature was set to 35 °C; the mobile phase consisted of acetonitrile containing 0.5% (*w*/*v*) aqueous phosphoric acid (98:2, *v*/*v*); isocratic elution mode was used; the flow rate was maintained at 1.0 mL/min; UV detection was performed at a wavelength of 208 nm; and the injection volume was 10 μL.

#### 3.2.4. Gas Chromatography Conditions

For gas chromatography, a DB-WAX quartz capillary column (30 mm × 0.32 mm × 0.5 m) was used. The starting column temperature was 200 °C, which was maintained for 26 min, before the temperature was raised by 5 °C/min to 220 °C, and maintained for an additional 10 min. The column flow rate was 1.5 mL/min, the inlet temperature was set to 250 °C, and the hydrogen flame detector was set to 250 °C. The injection volume was 1.0 μL, and the sample volume was 1.0 μL; the split ratio was 2:1, with high-purity nitrogen as the carrier gas.

### 3.3. In Vitro Antioxidant Capacity Assays

The biological activity of *P. utilis* seed oil extracts was evaluated using the four assays described below.

#### 3.3.1. 2,2-Diphenyl-1-picrylhydrazyl (DPPH) Radical Scavenging Assay

DPPH serves as a stable free radical with maximum absorbance between 515 nm and 517 nm. As DPPH interacts with hydrogen-donating substances like antioxidant compounds, it is reduced to its related hydrazine, resulting in no absorbance. Lower absorbance levels in the reaction mixture indicate higher free radical scavenging activity. The DPPH free radical scavenging activity of the samples was assessed following the method of Cao et al., with minor modifications [38].

Briefly, 0.1 mL of a 0.2 mM DPPH solution and 0.1 mL of each *P. utilis* seed oil sample were mixed in the wells of a 96-well plate. Ascorbic acid (5, 10, and 20 mg/mL) served as the positive control. The mixture was held at 20 °C for 30 min in the dark. For the blank samples, absolute ethanol was added instead of the *P. utilis* seed oil sample. The total volume of the assay was 200 µL. Absorbance was measured at 517 nm using a UV–visible spectrophotometer at the end of the reaction. Each determination was carried out in triplicate (*n* = 3). The radical scavenging activity (RSA) of the samples was calculated using the following formula:

RSA (%) = [1 − (A_1_ − A_2_)/A_0_] × 100% (1)

where A_1_ represents the absorbance of the sample reaction mixture, A_2_ is the absorbance of the blank (obtained by mixing 100 µL of each *P. utilis* seed oil sample with 100 µL of ethanol), and A_0_ is the absorbance of a control (obtained by mixing 100 µL of a 0.2 mM DPPH ethanol solution with 100 µL of ethanol). Ascorbic acid served as both an antioxidant standard and a positive control. Data are presented as the mean percentage of triplicate measurements ± standard deviation (SD).

#### 3.3.2. ABTS^+^ Radical Scavenging Assay

The ABTS^+^ free radical scavenging capacity test was carried out as described previously [38], with minor modifications. A stock solution of the ABTS radical cation (ABTS^+^) was prepared by combining a freshly made ABTS solution (7.682 mg/mL in water) with K_2_S_2_O_8_ (1.223 mg/mL) and incubating the mixture overnight (for at least 16 h) in the dark at room temperature. A working solution was then prepared by diluting the above stock solution with ethanol until an absorbance at 754 nm of 0.700 ± 0.05 was reached. For the assay, 160 µL of the ABTS^+^ working solution and 40 µL of each *P. utilis* seed oil sample (5, 10, and 20 mg/mL) were added to the wells of a 96-well plate. The mixture was incubated in the dark for 30 min. Gallic acid was used as a positive control, and the blank consisted of an equivalent volume of ethanol instead of the sample. The total volume of the assay was 200 µL. After incubation, absorbance was measured at 734 nm using an Infinite M200 microplate reader (Tecan, Grödig, Austria). All determinations were performed in triplicate (*n* = 3). RSA was calculated using the following equation:

RSA (%) = [1 − (A_3_ − A_4_)/A_5_] × 100% (2)

where A_3_ is the absorbance of the sample after incubation, A_4_ is the absorbance of the blank (obtained by mixing 40 µL of a *P. utilis* seed oil sample with 160 µL ethanol), and A_5_ is the absorbance of the negative control (obtained by mixing 160 µL of an ABTS^+^ solution with 40 µL ethanol). Ascorbic acid was used as the antioxidant standard and a positive control.

#### 3.3.3. Hyaluronidase Inhibition Assay

The hyaluronidase (HAase) inhibition assay was performed following the method described by Wang et al. [39], with trisodium glycyrrhizinate serving as the positive control. Fresh HAase was prepared in acetate buffer (pH 5.6). The *P. utilis* seed oil samples were diluted in DMSO to obtain concentrations of 5, 10, and 20 mg/mL. For the assay, 40 µL of HAase was added to each well of a 96-well plate, followed by 40 µL of the diluted *P. utilis* seed oil samples. The plate was incubated at 37 °C for 20 min. Subsequently, 8 µL 2.5 mol/L CaCl_2_ was added, and the plate was maintained at 37 °C for another 20 min. Then, 40 µL of substrate, 40 µL distilled water, 8 µL 5 mol/L sodium hydroxide, and 40 µL acetylacetone were added and mixed, and the mixture was kept in a boiling water bath for 15 min, then cooled in an ice bath for 10 min. Finally, 80 µL of p-phenylenediamine dihydrochloride (P-DAB) was added. For the sample control, HAase was replaced with an equivalent volume of acetate buffer. In the blank control and model control groups, an equivalent volume of water was used in place of samples; the model control group contained HAase, whereas the blank control group did not. The reaction with hyaluronic acid generates turbidity, and the decrease in turbidity was measured at 530 nm using an Infinite M200 microplate reader (Tecan, Austria). The percentage of HAase inhibition was calculated using the following equation:

HAase inhibition rate (%) = [(B − C) − (A − D)]/(B − C) × 100%(3)

where A is the absorbance of the sample, B is the absorbance of the model control, C is the absorbance of the blank control, and D is the absorbance of the sample control.

#### 3.3.4. Elastase-Inhibitory Activity Assay

Elastase-inhibitory activity was assessed spectrophotometrically using a modified version of established methods [31]. N-succinyl-Ala-Ala-Ala-p-nitroanilide (Suc-Ala-Ala-Ala-pNA; Sigma-Aldrich, St. Louis, MO, USA) was used as the substrate, and the quantity of released p-nitroaniline was determined at 415 nm using an Infinite M200 microplate reader (Tecan, Austria). The reaction mixture was prepared by adding 25 μL 10 mM Tris-HCl (pH 7.0–9.0) to each well, followed by 25 μL of the sample. Then, 50 μL of 2 mM Suc-Ala-Ala-Ala-pNA (prepared in 10 mM Tris-HCl, pH 8.8) was added. The mixture was allowed to react at 37 °C for 20 min, after which the absorbance was measured at 415 nm. One enzymatic unit was defined as the amount of elastase that releases 1 μmol of p-nitroaniline per min under the assay conditions. The percentage of elastase inhibition was calculated using the following formula:

Elastase-inhibitory rate (%) = 1 − [(A_2_ − A_1_)/(A_3_ − A_0_)] × 100 (4)

where A_0_ and A_1_ are the absorbance values of the blank and the sample control without enzyme, respectively; A_2_ and A_3_ are the absorbance values of the test sample and the model control with enzyme, respectively.

### 3.4. In Vivo Zebrafish Restoration Model

Zebrafish embryos were anesthetized for wounding in a 40.9 μg/mL tricaine methanesulfonate solution to minimize stress and movement. A full-thickness wound was carefully created in the caudal fin of each anesthetized embryo under a microscope using an experimental scalpel. The tailless zebrafish embryos were treated with 0.2 mL (CO:1%; RO:1%, 3%, 5%, 7%) oil samples of *P. utilis* in a 96-well plate at 28 ± 1 °C for 48 h. For the model control, 0.2 mL of fish embryo culture medium was used instead of the treatment solution. Post-treatment, the zebrafish were anesthetized again in a tricaine solution and photographed using a microscope. To quantify inflammation, entire wounds were photographed using identical settings at the indicated time points. The promotion rate of caudal fin repair was calculated using the following formula:

Promotion rate (%) = (S − C)/C ×100%(5)

where S and C are the lengths of the caudal fins of zebrafish embryos in the subject treatment and the model control, respectively.

The model control group consisted of fish embryo culture medium; the control group was a Rehmannia glutinosa extract.

### 3.5. Statistical Analysis

All experimental results are expressed as the arithmetic mean ± standard deviation (SD) from three independent replications. Statistical analysis was performed using IBM SPSS Statistics version 26.0 software (SPSS, Chicago, IL, USA). For assays with two independent variables (sample type and concentration), two-way analysis of variance (ANOVA) was applied to evaluate the main effects of each factor and their interaction effects, followed by Duncan’s multiple range post hoc test for pairwise comparisons when a significant interaction was detected. For assays with a single independent variable, one-way analysis of variance (ANOVA) followed by Tukey’s multiple comparison test was used to evaluate differences between groups. Differences were considered statistically significant at *p* ≤ 0.05, while 0.05 < *p* ≤ 0.10 was interpreted as a trend that did not reach significance.

## 4. Conclusions

In this study, we performed a phytochemical profiling of *P. utilis* seed oil using UPLC—QTOF MS/MS, and tentatively annotated 14 discriminatory metabolites in CO and RO samples prepared by two processing methods. Furthermore, we evaluated the biological activities of CO and RO samples by determining their ability to scavenge DPPH and ABTS^+^ free radicals, measuring their inhibition of Haase and elastase, and examining the regeneration rates of amputated zebrafish fins in vivo. The identities of these bioactive compounds are highly consistent with the biological activities exhibited by *P. utilis* seed oil. The results of this study support that CO is more used for functional foods with antioxidants and anti-aging activities, while RO is better suited for pharmaceuticals and cosmetics, depending on two different processing methods. Refining CO to obtain RO altered the relative amounts of sterols such as campesterol glucoside and elevated the contents of fatty acids such as 17-octadecynoic acid, as well as terpenoids such as petasin and sterebin D. These tentatively annotated compounds have been reported to exhibit anti-inflammatory, antioxidant, anti-aging, antibacterial, moisturizing, and photoprotective properties in previous studies. Chemometric analysis revealed that two processing methods affected the bioactive compound content of the oil, with CO and RO demonstrating HAase and elastase inhibition in vitro, and RO presents higher antioxidant capacity and significant promotion of caudal fin regeneration in vivo than CO. We conclude that *P. utilis* seed oil is a promising source of tocopherol, squalene, sterols, fatty acids, and other bioactive compounds with food-medicine homology in health and wellness under different processing methods. Our findings elucidate the functional compounds in *P. utilis* seed oil and highlight its potential for development as a natural raw material for medicine and food of homological application as well as cosmetics, offering initial validation of its traditional applications.

## Data Availability

All data are described in the main text and figures and can be made available upon request.

## Supplementary Materials

The following supporting information can be downloaded at: https://www.mdpi.com/article/10.3390/molecules31101700/s1, Figure S1. Tocopherol contents in CO samples. Figure S2. Squalene in CO samples. Figure S3. Sterol content in CO samples. Figure S4. Gas chromatography analysis of fatty acids in CO samples. Table S1. Tentatively annotated differential metabolites between crude oil (CO) and refined oil (RO) of *P. utilis* seed oil (VIP > 1, *p* < 0.05). Table S2. Two-way ANOVA for DPPH radical scavenging rate as a function of sample type and concentration. Table S3. Two-way ANOVA for ABTS^+^ radical scavenging rate as a function of sample type and concentration. Table S4. Two-way ANOVA for hyaluronidase (HAase) inhibition rate as a function of sample type and concentration. Table S5. Two-way ANOVA for elastase inhibition rate as a function of sample type and concentration.
