# Supplementary material for: Bioactive Compounds and Related Food-Medicine Homology Potential of Prinsepia utilis Seed Oil
Source: Molecules. 2026 May 17;31(10):1700. doi: 10.3390/molecules31101700 (PMC13209670; doi:10.3390/molecules31101700)
Supplement: Supplementary file 1 [file molecules-31-01700-s001.zip › Supplementary File S2.pdf]

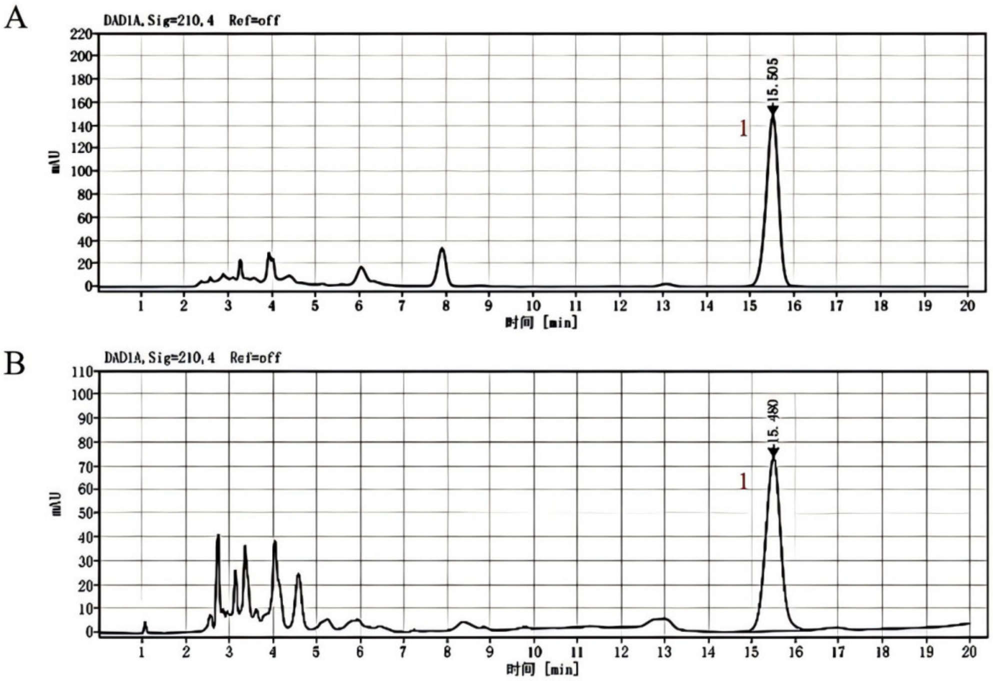

Figure S2. Squalene in CO samples. (A) Representative chromatogram for the squalene standard by HPLC. (B) Determination of squalene in CO samples by HPLC. 1, squalene.
