# Supplementary material for: Bioactive Compounds and Related Food-Medicine Homology Potential of Prinsepia utilis Seed Oil
Source: Molecules. 2026 May 17;31(10):1700. doi: 10.3390/molecules31101700 (PMC13209670; doi:10.3390/molecules31101700)
Supplement: Supplementary file 1 [file molecules-31-01700-s001.zip › Supplementary File S3.pdf]

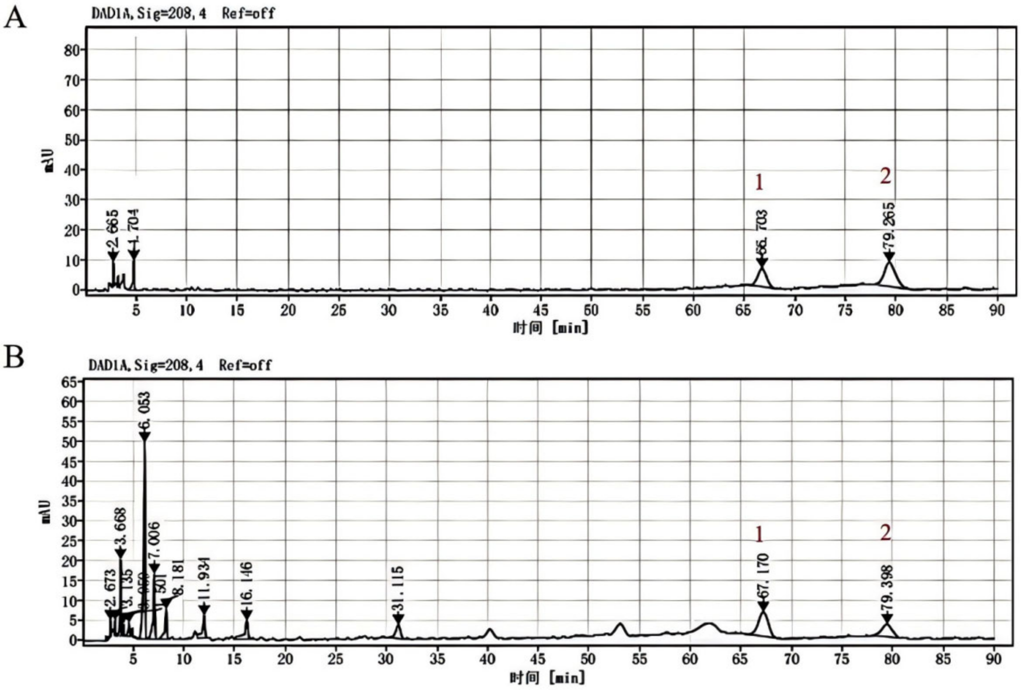

Figure S3. Sterol content in CO samples. (A) Representative chromatogram of the sterol standard by HPLC. (B) Determination of sterols in crude oil samples by HPLC. 1, stigmasterol; 2, β-sitosterol.
