# Supplementary material for: Bioactive Compounds and Related Food-Medicine Homology Potential of Prinsepia utilis Seed Oil
Source: Molecules. 2026 May 17;31(10):1700. doi: 10.3390/molecules31101700 (PMC13209670; doi:10.3390/molecules31101700)
Supplement: Supplementary file 1 [file molecules-31-01700-s001.zip › Supplementary File S4.pdf]

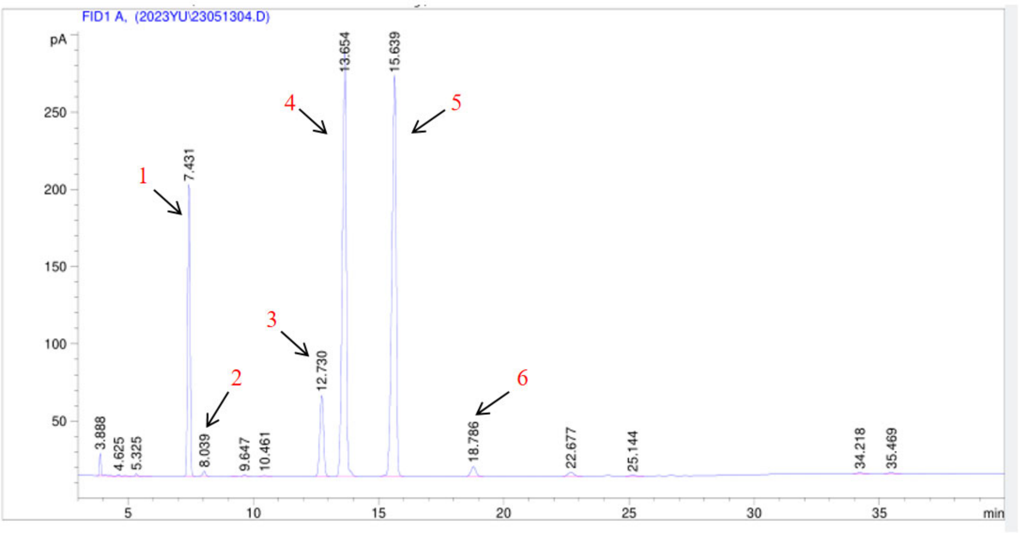

Figure S4. Gas chromatography analysis of fatty acids in CO samples. 1, Palmitic acid (C16:0); 2, palmitoleic acid (C16:1); 3, stearic acid (C18:0); 4, oleic acid (C18:1); 5, linoleic acid (C18:2); 6, linolenic acid (C18:3).
