# Supplementary material for: Bioactive Compounds and Related Food-Medicine Homology Potential of Prinsepia utilis Seed Oil
Source: Molecules. 2026 May 17;31(10):1700. doi: 10.3390/molecules31101700 (PMC13209670; doi:10.3390/molecules31101700)
Supplement: Supplementary file 1 [file molecules-31-01700-s001.zip › Supplementary Table S(1-5).pdf]

Supplementary Result

The initial metabolite annotation list was conservatively re-curated to remove database-derived but oil matrix-incompatible assignments. The current Supplementary Table S1 only includes chemically plausible, tentatively annotated metabolite features. Metabolites were retained in Supplementary Table S1 based on three primary criteria: compatibility with natural plant origin, lipid solubility matching the oil matrix, and reliable MS/MS spectral interpretability.

Table S1

Tentatively annotated differential metabolites between crude oil (CO) and refined oil (RO) of *P. utilis* seed oil (VIP > 1, $p < 0.05$)

No.	Compound	VIP	*P*
16.39_280.2402n	17-Octadecynoic acid	1.778265	0.0000056072
6.56_295.2265m/z	Hydroxyoctadecadienoic acid	1.755288	0.00022863
13.67_371.3156m/z	Heneicosanoic acid	1.775288	0.000016519
17.73_423.4191m/z	Octacosanoic acid	1.76728	0.000077022
19.32_309.2426m/z	Octadeca-9,12-dienal	1.762429	0.000092742
23.75_367.3574m/z	Tetracosanoic acid	1.7476	0.00071173
16.39_311.2216m/z	18-Hydroperoxyoctadeca-2,4-dienoic acid	1.748505	0.00059724
16.39_293.2112m/z	Sterebin D	1.776871	0.0000068966
16.39_379.1576m/z	Petasin	1.777415	0.0000033494
17.03_211.1330m/z	Vulgarole	1.744838	0.00047123
16.39_307.2272m/z	6,10,14-Trimethyl-5,9,13-pentadecatrien-2-one	1.779978	0.0000020698
16.39_225.1487m/z	4,5-Dihydrovomifoliol	1.779831	0.0000025041
19.55_607.4190m/z	Campesterol glucoside	1.74805	0.0004111
16.38_861.6772m/z	Coenzyme Q10	1.770519	0.00002383

Note: All metabolites listed are tentatively annotated based on plant origin compatibility, lipid solubility matching the oil matrix, and reliable MS/MS spectral interpretability. No database-derived, oil matrix-incompatible annotations were included.

Table S2 Two-way ANOVA for DPPH radical scavenging rate as a function of sample type and concentration

Source	df	SS(U^2/g^2)	MS(U^2/g^2)	F	p-Value	Partial η^2
Sample type	2	29287.788	14643.894	2764.966	<0.001	0.997
Concentration	2	1381.646	690.823	130.437	<0.001	0.935
Sample type × Concentration	4	2040.424	510.106	96.315	<0.001	0.955
Error	18	95.332	5.296	-	-	-

Table S3 Two-way ANOVA for $ABTS^+$ radical scavenging rate as a function of sample type and concentration

Source	df	SS(U^2/g^2)	MS(U^2/g^2)	F	p-Value	Partial η^2
Sample type	2	35369.719	17684.859	5202.517	<0.001	0.998
Concentration	2	1423.422	711.711	209.371	<0.001	0.959
Sample type × Concentration	4	2012.723	503.181	148.025	<0.001	0.970
Error	18	61.187	3.399	-	-	-

Table S4 Two-way ANOVA for hyaluronidase (HAase) inhibition rate as a function of sample type and concentration.

**Source**	**df**	**SS(U^2/g^2)**	**MS(U^2/g^2)**	**F**	**p-Value**	**Partial η²**
Sample type	2	4394.171	2197.085	152.860	<0.001	0.944
Concentration	2	2429.476	1214.738	84.514	<0.001	0.904
Sample type × Concentration	4	126.521	31.630	2.201	0.110	0.328
Error	18	258.718	14.373	-	-	-

Table S5 Two-way ANOVA for elastase inhibition rate as a function of sample type and concentration.

**Source**	**df**	**SS(U^2/g^2)**	**MS(U^2/g^2)**	**F**	**p-Value**	**Partial η²**
Sample type	2	2741.344	1370.672	23.974	<0.001	0.727
Concentration	2	3980.658	1990.329	34.813	<0.001	0.795
Sample type × Concentration	4	698.283	174.571	3.053	0.044	0.404
Error	18	1029.103	57.172	-	-	-
